# Cardiac Paraganglioma in a Young Patient Presents with Angina-like Symptoms: A Case Report and Literature Review

**DOI:** 10.3390/medicina60091495

**Published:** 2024-09-13

**Authors:** Batool Wael Alnahar, Bushray Almiqlash, Hala Hassanain, Ebtesam Al-Najjar, Abdullah Esmail, Asma Zainab, Iqbal Ratnani

**Affiliations:** 1 Division of Cardiovascular ICU, Department of Critical Care Medicine, Houston Methodist Hospital, Houston, TX 77030, USA; 2 Department of Cardiothoracic Surgery, Cincinnati Children’s Hospital, Cincinnati, OH 45229, USA; 3 Department of Medicine, Houston Methodist Hospital, Houston, TX 77030, USA; 4 Section of GI Oncology, Houston Methodist Neal Cancer Center, Houston Methodist Hospital, Houston, TX 77030, USA

**Keywords:** paraganglioma, cardiac tumor, neuroendocrine tumor, catecholamines, surgical excision, diagnostic imaging, adult cardiac tumors

## Abstract

Paragangliomas are rare extra-adrenal neuroendocrine tumors originating from chromaffin tissue that present a diagnostic and therapeutic challenge due to their diverse clinical manifestations and low incidence. While these tumors often manifest as catecholamine-secreting functional tumors, their clinical presentation can vary, leading to delayed diagnosis and challenging management. This study presents the case of a 22-year-old patient with cardiac paraganglioma who initially presented with angina-like symptoms, highlighting the importance of considering this rare condition in young individuals with nonspecific complaints. Diagnostic imaging, including transthoracic echocardiography, CT angiography, and MRI, played a crucial role in identifying the tumor’s location and vascularization. Surgical excision, including pulmonary artery graft and CABG, was the primary management approach, which was accompanied by intraoperative complications that later led to CCU admission, followed by postoperative complications, ultimately leading to the patient’s death. This case highlights the significance of early recognition and management of complications following a surgical approach to treat paragangliomas.

## 1. Introduction

Paragangliomas are extra-adrenal neuroendocrine tumors originating from the chromaffin tissue of the sympathetic and parasympathetic nerve chains [1]. These tumors are rare, with an incidence of 2–8 per million per year [2]. While the organ of Zuckerkandl is the most frequent location for paragangliomas, they can also occur in autonomic nerve chains of the abdomen, chest, or bladder [2,3]. Cardiac paragangliomas are considered extremely rare, accounting for less than 1% of primary cardiac tumors, which are most frequently found in the left atrium. Most patients are diagnosed between the fourth and sixth decades of their lives [4]. They are classified based on their secretion of catecholamine into functional and nonfunctional tumors [5]. Episodic headaches, palpitations, and sweating are the characteristic features of catecholamine-secreting tumors. The intermittent nature of catecholamine release in paraganglioma can lead to a delayed diagnosis [6]. Dyspnea is one of the symptoms that can be triggered by the compression effect of the tumor or by its excess secretion of catecholamines. Although tumor resection is reported as the most essential treatment for cardiac paraganglioma, the 5-year overall survival is only 78.8% for those with benign tumors and even lower for others with malignant tumors [7]. This poor prognosis could be explained by a lack of disease awareness and delayed diagnosis. Therefore, early proper diagnosis of the tumor is essential to decrease the disease-related complications and mortality rate [8]. In this study, we report a case of a very young patient with cardiac paraganglioma who initially presented with angina-like symptoms, prompting our study to understand the possible presentations of this disease better.

## 2. Case Presentation

A 22-year-old woman was admitted to the hospital following a six-month history of progressively worsening episodes of exertional dyspnea and chest pain. Initially, her symptoms were thought to be related to an anxiety disorder, and she was started on sertraline and buspirone, which relieved her anxiety symptoms but had no relieving effect on the dyspnea or the chest pain. The patient described the pain as starting in the chest area, with radiation to the left arm, jaw, and associated with nausea. The patient reported smoking one pack of cigarettes daily. On physical examination, she had sinus tachycardia, blood pressure (BP) of 121/66 mmHg, and her heart rate was 104 beats/min. No murmur was heard in the precordium and no edema was found in the extremities. As her symptoms continued to worsen over a period of one month, she underwent an extensive workup to reach a definitive diagnosis, following the discontinuation of her anxiety medication.

**Figure 1:**
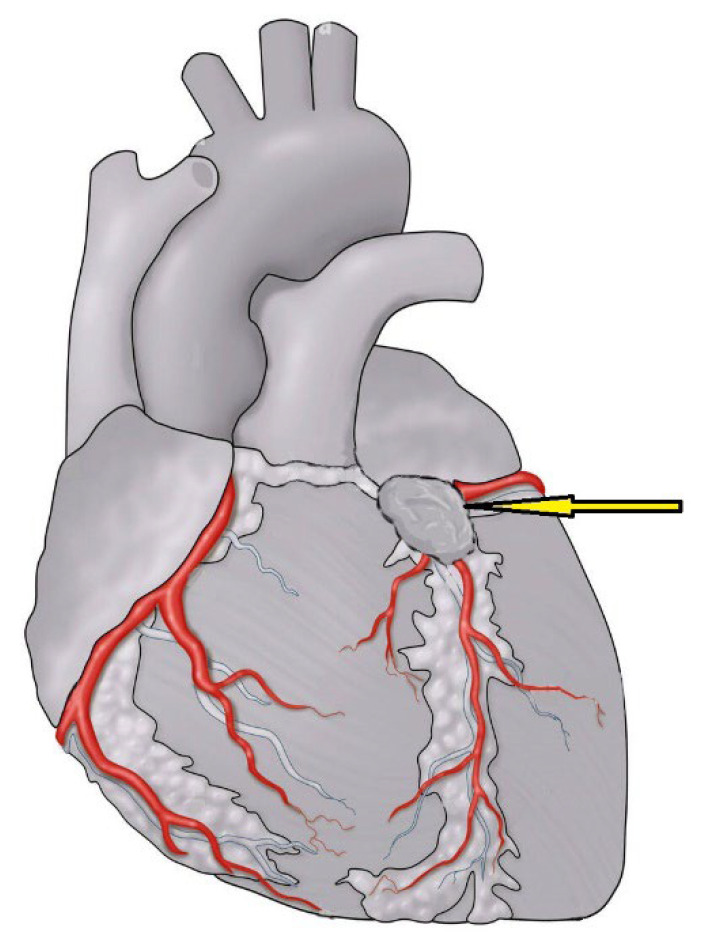
Anatomical location of the tumor showed a highly vascularized mass (size: 7.5 × 4.1 cm) encasing the LAD and the proximal LCX.

Transthoracic echocardiography (TTE) was performed, revealing a large mediastinal mass on the anterolateral surface of the left atrium (LA) and left ventricle (LV) reaching up to the proximal aorta (Figure 1). The mass was found almost encasing the left main coronary artery origin and the proximal pulmonary artery (PA). Furthermore, the bifurcation of the left anterior descending artery (LAD) and the left circumflex artery (LCX) was visualized inside the mass. The echocardiography also revealed a turbulent flow across the right PA. However, the bi-ventricular systolic and diastolic functions were normal.

**Figure 2:**
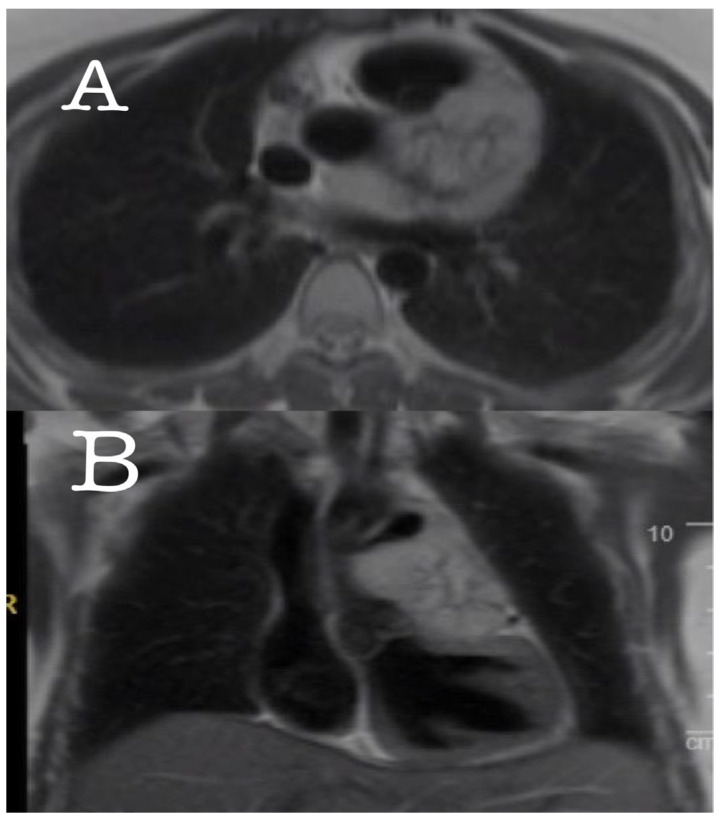
Axial (**A**) and coronal (**B**) MRI showed a mass encasing the LAD and the proximal LCX.

**Figure 3:**
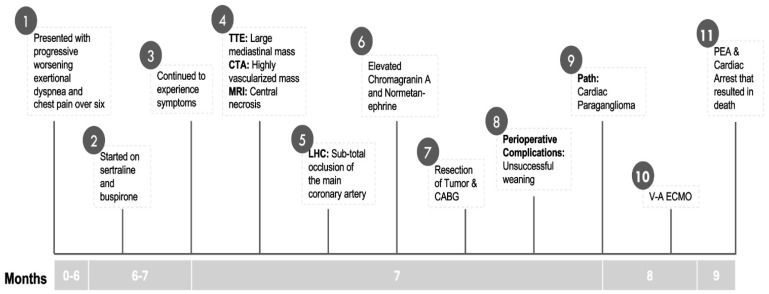
Detailed timeline emphasizing the case history.

For further details, a computerized tomography (CT) angiography was performed, which showed a highly vascularized mass (size: 7.5 × 4.1 cm) encasing the LAD and the proximal LCX. Magnetic resonance imaging (MRI) of the chest also revealed an area of central necrosis within the mass (Figure 2 and Figure 3). Moreover, a left heart catheterization was performed and showed a sub-totally occluded left main coronary artery with 90% obstructed LCX. Laboratory investigations revealed an increased serum level of normetanephrine (3.21 nmol/L, normal range is less than 0.9 nmol/L) with a high chromogranin A level (859 nmol/L, normal range 0–6.0 nmol/L), which were suggestive of a paraganglioma tumor. The genetic was performed and resulted as positive for mutations in the succinate dehydrogenase gene (SDHx); one pathogenic variant, c.725G>A; p.Arg242His, was detected in the SDHB gene by massively parallel sequencing. This result is consistent with a diagnosis of hereditary paraganglioma–pheochromocytoma syndrome; clinical manifestations are variable. This individual’s offspring have a 50 percent chance of inheriting the pathogenic variant.

Based on these findings, the patient underwent a procedure to resect the tumor and reconstruct the PA using a cadaver graft. Additionally, a coronary artery bypass graft (CABG) was performed using a saphenous vein graft to obtuse the marginal (OM) coronary artery along with another CABG using the left internal mammary artery to construct the LAD. Preoperatively, the patient was given a 500 mL lactated Ringer’s solution with 0.5 mg of doxazosin. During the procedure, the patient was on cardiopulmonary bypass (CPB) and was shocked twice due to ventricular fibrillation and ventricular tachycardia. Postoperatively, weaning off the CPB was unsuccessful, which led the patient to be brought to the cardiovascular intensive care unit (ICU) while intubated, under sedation, and later the patient was put on central venous–arterial extracorporeal membrane oxygenation (ECMO).

Pathological examination of the tumor exhibited nests of tumor cells located in fibrovascular septa with scattered cells showing prominent nucleoli. The immunohistochemistry revealed that tumor cells were positive for synaptophysin, chromogranin, and CD56, and negative for the cytokeratin cocktail. S100 stain highlighted scattered sustentacular cells. The findings were consistent with a histological diagnosis of cardiac paraganglioma.

After admission to the cardiovascular ICU on central venous–arterial ECMO, the patient developed acute postoperative respiratory failure, which was managed with assisted–controlled synchronized intermittent ventilation. Moreover, her postoperative echocardiography showed a decrease in the LV ejection fraction (25%), hypokinesia in the anterior and inferior walls, and moderately depressed right ventricular function. Consequently, the patient developed a cardiac arrhythmia that did not respond to CPR and eventually died within months after she initially presented.

## 3. Discussion

The presented case highlights the intricate nature of cardiac paragangliomas which are characterized by diverse clinical manifestations. In this instance, a 22-year-old woman with a history of smoking presented with progressively worsening exertional dyspnea and chest pain. Initially, her symptoms were attributed to anxiety disorder, highlighting the potential diagnostic challenges posed by these tumors. A similar diagnostic journey was observed in the cases described by Chung et al., 2020 [9], Tahir et al., 2009 [10], and Liu et al., 2022 [11], where patients initially presented with symptoms that led to alternative clinical considerations. This reinforces the importance of a high index of suspicion and a comprehensive diagnostic approach when evaluating patients with symptoms that may be indicative of cardiac paragangliomas.

In the prospective observational study by Geroula et al., the aim was to determine whether specific symptoms or clinical signs observed in patients suspected of having pheochromocytomas and sympathetic paragangliomas (PPGLs) could indicate a higher or lower likelihood of the condition [12]. Patients were tested for PPGLs due to signs and symptoms, incidental masses found on imaging, or routine surveillance based on previous history or hereditary risk. On follow-up, 245 patients with PPGLs were identified, compared to 1820 without. Geroula et al. reported that hyperhidrosis, palpitations, pallor, tremor, and nausea were 30–90% more prevalent (*p* < 0.001) among patients with PPGLs compared to those without. In contrast, headache, flushing, and other symptoms showed little or no difference. Although heart rates were significantly higher (*p* < 0.0001) in patients with PPGLs, blood pressures were not higher and were positively correlated with BMI, which was lower (*p* < 0.0001) in these patients. Based on these clinical differences, a scoring system was developed, which indicated a 5.8-fold higher probability of PPGLs in patients with high scores compared to those with low scores. Higher scores among patients with PPGLs were associated, independently of tumor size, with elevated biochemical indices of catecholamine excess [12].

Martucci et al. [13] conducted a retrospective analysis of 15 patients with cardiac paragangliomas to investigate their clinical genetic background. Of the 13 patients (86.7%) who underwent genetic test, 10 (76.9%) was found to have mutations in SDHx subunits B, C, or D. All 13 patients (86.7%) who had surgery to remove the paraganglioma experienced no intraoperative morbidity or mortality. This study aligns with our case, which also tested positive for the SDHx mutation [12].

A study published by Eisenhofer et al. [14] emphasized that pheochromocytomas are significant risks due to their secretion of large amounts of vasoactive catecholamines, which can lead to high blood pressure and related complications. These tumors are often undiagnosed until therapeutic interventions trigger catecholamine release. Because they are rare, evidence against certain drugs is largely anecdotal. Some medications, such as dopamine D2 receptor antagonists (e.g., metoclopramide) and β-blockers, have a high risk of adverse reactions, while others, like tricyclic antidepressants, are less consistent in causing issues. Additionally, drugs like monoamine oxidase inhibitors, sympathomimetics (e.g., ephedrine), and certain hormones (e.g., corticotropin) can also cause adverse reactions. To minimize risks, appropriate precautions can be taken, such as adrenoceptor blockade. Without these safeguards, cardiovascular vulnerability during surgical anesthesia is heightened. Problems often occur when medications or procedures are used in patients without a suspected tumor. Therefore, clinicians must be alert to the possibility of a catecholamine-producing tumor and manage adverse events accordingly. Based on these, our doctors decided to discontinue the anxiety medication for a period before surgery.

Our patient was positive for mutations in the SDHx. This result is consistent with a diagnosis of hereditary paraganglioma–pheochromocytoma syndrome; clinical manifestations are variable. This individual’s offspring have a 50 percent chance of inheriting the pathogenic variant

Advanced imaging techniques, including echocardiography, CT, and MRI, serve as crucial diagnostic tools for assessing the location, size, and involvement of adjacent structures in cardiac paragangliomas. These modalities enable precise surgical planning, ensuring the best possible outcomes. Notably, Tahir et al., 2009 [10], presented a 37-year-old patient with substernal chest pain, and Chung et al., 2020 [9], reported a 39-year-old man with chest pain, heart palpitation, and hypertension. Conversely, Liu et al., 2022 [11], detailed the case of a 67-year-old woman presenting with palpitations, fatigue, and lower extremity edema. However, the complexities of these tumors, as revealed by Liu et al., 2022 [11], can pose significant challenges during surgery, underscoring the importance of preoperative assessment and planning.

Surgical management of cardiac paragangliomas requires meticulous planning, often requiring CPB due to the tumor’s intricate location around critical cardiac structures. In their 2021 systematic review, Fang et al. [15] discussed that the main goal of preoperative management for PPGLs is to normalize blood pressure and heart rate. The standard approach involves blocking excessive plasma catecholamines, with α-adrenergic receptor (α-AR) antagonists being the preferred first choice. Non-selective α-AR antagonists, such as phenoxybenzamine, may provide slightly better control of hypertension for some patients, but they come with a higher risk of postoperative hypotension and other side effects. In contrast, selective α-AR antagonists like doxazosin have fewer adverse effects but are often used in combination with additional antihypertensive medications. The use of β-adrenergic receptor (β-AR) antagonists depend on the severity of catecholamine-induced tachycardia or reflex tachycardia following phenoxybenzamine administration. It is important to note that β-AR antagonists should not be used alone or before adequate α-AR blockade. To enhance blood pressure control in PPGL patients, calcium channel blockers (CCBs) are frequently administered alongside α-AR antagonists. Some studies suggest that CCB might be the preferred option for preoperative management, especially for normotensive patients, those with very mild hypertension, or patients who experience severe side effects with α-AR antagonists. For patients with severe symptoms that are not well controlled by other medications, such as those with biochemically active or extensively metastatic tumors, metyrosin, a catecholamine synthesis inhibitor, is used in combination with α-AR antagonists. A thorough cardiovascular evaluation is essential for all PPGL patients, and it is recommended to include a high-sodium diet and increased fluid intake in the treatment plan to restore blood volume preoperatively and prevent perioperative hypotension.

Araujo-Castro et al. [16] conducted a retrospective study in 2024 that analyzed 296 surgeries for PPGL. The study revealed that α-AR antagonists presurgery were used in 93.2% of cases, while β-AR antagonists were used in 53.4% of cases. Hypertensive crisis occurred in 20.3% of the surgeries, with 56 cases being intraoperative and 6 postoperative. The study identified several risk factors for intraoperative hypertension crisis, including the absence of presurgical glucocorticoid therapy, larger tumor size, and lack of oral sodium repletion. The author concluded that patients with higher presurgical systolic blood pressure who did not receive oral sodium repletion or were not pretreated with glucocorticoid therapy are at higher risk for developing hypertensive crisis during surgery.

Ohmachi et al. [17] published a retrospective study involving 51 patients who underwent surgical resection of PPGL. The study compared two groups: 14 patients who received doxazosin at maximum doses in combination with metyrosine and 37 patients who received doxazosin alone. The author highlighted that using metyrosine in combination with doxazosin as a preoperative treatment significantly affects intraoperative circulatory hemodynamics, notably reducing the incidence of blood pressure elevation during surgery.

A retrospective study of 19 patients with cardiac paragangliomas reported operative and 30-day hospital mortality of 10.6% with significant associated operative morbidity. This included cardiac tamponade, transfusions in 74% of cases, respiratory failure requiring tracheostomy, and mechanical cardiac support [18]. Chung et al., 2020 [9], and Tahir et al., 2009 [10], described successful surgical interventions guided by comprehensive imaging evaluations, which led to favorable outcomes. In our and Tahir et al.’s, 2009 [10], cases, the patients underwent CABG in addition to tumor resection, reflecting the complex nature of surgical management. While successful resection and reconstruction can result in positive outcomes, complications, as seen in Tahir et al., 2009 [10], can affect the postoperative course. Additionally, the histopathological confirmation of paraganglioma through immunohistochemistry is a critical step in guiding treatment and postoperative monitoring. Overall, these cases collectively demonstrate the complex nature of cardiac paragangliomas, emphasizing the need for a multidisciplinary approach involving cardiology, surgery, and critical care to manage these rare cardiac tumors effectively.

## 4. Conclusions

Surgical excision remains the gold standard curative treatment, but its associated complications pose a significant mortality and morbidity risk to patients. Notably, these tumors can be functional, secreting catecholamines in a substantial percentage of cases, further complicating their clinical management. Preoperative imaging, specifically cardiac CT angiography or invasive angiography, is imperative to meticulously delineate tumor vascularization, particularly its relationship with the coronary arteries. Paragangliomas can receive perfusion from coronary arteries, necessitating additional surgical considerations, including potential coronary revascularization. In challenging cases where immediate surgical resection is hindered, measures such as CPB and preoperative embolization of feeding arteries can be invaluable in reducing intraoperative bleeding risk, as exemplified in this study.

## Data Availability

The data of this study that support our results are available on request from the corresponding author.
